# Optimization of Vascular Supply in Free Flaps for Head and Neck Reconstruction: Analysis of a Young Team’s Experience

**DOI:** 10.3389/fsurg.2022.912010

**Published:** 2022-06-30

**Authors:** Paolo Iacoviello, Susanna Bacigaluppi, Simone Callegari, Carlo Rossello, Andrea Antonini, Marco Gramegna, Mariano Da Rold, Giuseppe Signorini, Giuseppe Verrina

**Affiliations:** ^1^ Department of Maxillofacial and Plastic Reconstructive Surgery, E.O. Ospedali Galliera, Genoa, Italy; ^2^ Department of Neurosurgery, E.O. Ospedali Galliera, Genoa, Italy; ^3^ Department of Neurosurgery and Neurotraumatology, IRCCS Policlinico San Martino, Genoa, Italy; ^4^ DINOGMI, Neurosurgery and Neurotraumatology, University of Genoa, Genoa, Italy; ^5^ Burn Unit and Plastic Surgery, Villa Scassi Hospital, Genoa, Italy; ^6^ Regional Center of Hand Surgery, Savona, Italy; ^7^ MIOS S. Maria Misericordia Hospital, Albenga, Italy

**Keywords:** head and neck reconstruction surgery, microsurgery, free flap, recipient vessel for free flap transfer, flap survival, flap re-exploration

## Abstract

**Background:**

For head and neck reconstructive procedures, free flap survival depends on microsurgical and anatomical choices besides multimodal clinical management. The aim of the present study is to identify relevant variables for flap survival in our initial consecutive series.

**Methods:**

A single-center, novel reconstructive team consecutive surgical series was revised. The outcome was analyzed in terms of flap survival observing variables considered more relevant: flap type, recipient artery, vein(s), and graft interposition were discussed for facial thirds to be reconstructed. Statistical analysis was performed with Chi-square, Mann–Whitney, and Odds ratio.

**Results:**

A total of 118 free flaps were performed in 115 microsurgical procedures (93.9% for malignancies) on 109 patients, with a flap survival rate of 91.5%. For reconstruction of the middle and lower third of the face, the facial artery was privileged, because it was already transected during lymph node dissection in order to save the superior thyroid artery for further microsurgical needs. Flap failure was 50% venous. Double vein anastomosis was not related to flap survival. Deep venous drainage (as the internal jugular vein system) required fewer revisions. Half of the re-explorations saved the flap. Grafts were a risk for flap survival. Bony flaps were more critical.

**Conclusion:**

At comparable reconstructive quality, flap choice should avoid a vascular graft. The facial artery is a preferable recipient vessel, since it saves other arteries both in the case of an arterial revision and in the case of recurrence, for further free flap reconstruction. For venous anastomosis, a deep venous recipient is safer, since it offers the possibility to choose the level of anastomosis optimizing the vascular pedicle geometry. A close postsurgical flap monitoring is advisable up to 7 days postoperatively to allow for timely flap salvage.

## Introduction

Microsurgical free flaps are a key tool for the reconstruction of complex defects in the head and neck region (1). The choice of the more appropriate flap to be tailored depends on the type of the defect and on the site to be reconstructed. Depending on the structures that have to be restored for functional and esthetic needs, the flap can be harvested either with a bony component or only with a combination of soft tissues (e.g., skin, muscle, fascia).

For reconstructive purposes, the face is conventionally divided into thirds: upper, middle, and lower. These regions offer multiple options for recipient vessel choice. The length of the arterial and of the venous flap pedicles, together with the distance between the anastomotic site and the defect to be covered, determines the range of possible options for safe revascularization.

As regards factors that contribute to successful flaps, there are several studies that deal with a single question only, but there are few studies that analyze different aspects. For example, there is no definitive clarity concerning single or double vein anastomosis for the free flap (2, 3). Therefore, the aim of our study was to analyze in our series the variables of recipient vessel choice that contributed to flap survival.

## Patients and Methods

We analyzed a retrospective database of a consecutive series of microsurgical free flaps of head and neck reconstructive procedures from July 2011 to February 2020 at Galliera Hospital in Genoa. The study was carried out in accordance with the Helsinki declaration of 1975 as revised in 1996.

Our database kept track of the patient’s gender, age, ASA score, site of the defect, and consequently, the facial thirds involved. We defined for reconstructive purposes the three facial thirds divided by two lines: the upper line runs from the orbital roof/eyebrow to the level of the helix; the inferior line from the lip commissure to the ear lobe. Furthermore, we collected all data about the diagnosis of the lesion to be removed; in case of malignant tumors, staging, grading with particular attention to lymph node involvement. We specified whether the lesion was either a primitive tumor or a recurrence/remnant and whether the patient had undergone presurgical radio- or chemotherapy.

**Figure 1:**
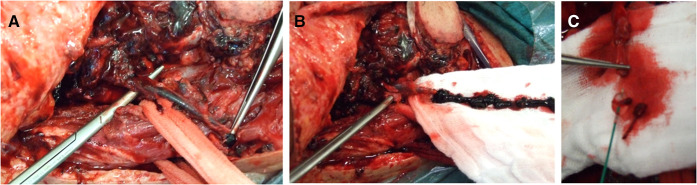
Venous flap revision. (**A**) Thrombotic vein detached from the anastomosis. (**B**) Vein squeezing and thrombectomy. (**C**) Subsequent mechanical thrombectomy by using the Fogarty catheter.

As regards our focus for the present study, we kept track of the type of free flap used to cover the defect. We specified the recipient artery used and the vein(s) chosen for anastomosis. We showed a special interest in the preparation of a double vein anastomosis. For the recipient vein, we differentiated between superficial and deep, considering that superficial anastomoses might be more exposed to inadvertent compression in the postsurgical stay—such as compression by the elastics of the Venturi mask, the tracheostomy tube holder, and the stick of the glasses. For double veins, we classified the flap as having a deep venous drainage if at least one recipient was deep. For our purposes, we defined as superficial drainage the external jugular vein (EJV) and obviously its retromandibular tract, the superficial temporal vein (STV), and the facial vein (FV) up from the mandibular bone. We defined as deep drainage the internal jugular vein (IJV) from end to side, the thyro-linguo-facial trunk (TLV), and the lingual vein (LV). We considered the TLV or LV as a deep drainage since we always performed anastomosis very closely to the IJV so that the end-to-end anastomosis resembled an end-to-side anastomosis to the IJV as regards pressure. We annotated where a vessel graft was required. Furthermore, we also marked when microvascular revision was required. We performed flap revision after flap monitoring: visual inspection, refill, and ultrasound performed every hour for the first 24 h and then every 2 h for 5 days. The suspected anastomosis was inspected at revision surgery with a patency test and the thrombosed anastomosis was sectioned. Endoluminal heparin flushing was performed for both arterial and venous recanalization. To manage arterial plugs, thrombokinases were also used to wash the flap, avoiding outflow going into the systemic circulation. For venous thromboses, the mechanical thrombectomy with a Fogarty catheter was used (Figure 1). Since our focus was flap survival, we created a file for each flap procedure. In our database, some patients needed more than one free flap procedure over time for retreatment of recurrence or for delayed reconstruction to improve the functional and esthetic outcome. Some patients underwent a double flap reconstructive procedure.

**Figure 2:**
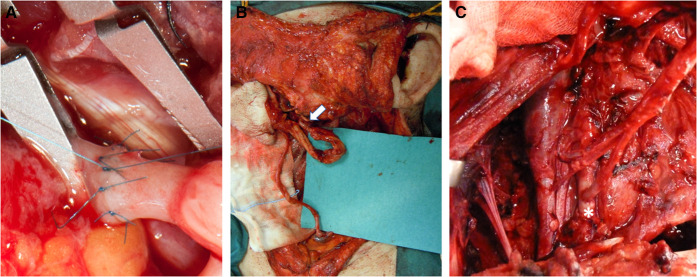
Arterial recipient vessels. (**A**) End-to-end arterial anastomosis detail. (**B**) Anastomosis between the transected supra digastric facial artery and the radial artery flap pedicle (white arrow). (**C**) End-to-side anastomosis to the external carotid artery (white asterisk).

Technically, we performed the anastomosis with separate stitches, with either 8.0 or 9.0 polypropylene monofilament (Figure 2A).

The features of the retrieved case series and of surgical procedures were summarized as mean with SD for continuous variables and percentages for dichotomous variables. The odds ratio and key square test were used to test the association of one surgery procedure/variable with the risk of flap failure (Prism 5, GraphPad software).

## Results

We performed 115 procedures with 118 free flaps on a total of 109 patients during July 2011 and February 2020 at Galliera Hospital in Genoa, Italy. In three patients, two free flaps were implanted using the same procedure.

### Patient Characteristics

Procedures were performed on 77 male and on 38 female patients, with a mean age at the procedure of 63 years (SD ± 13 years) and a range of 17–91 years. The mean ASA score of the patient at each procedure was 2.37 (SD ± 0.61), and the frequencies were 8 ASA1 (7%), 57 ASA2 (49.6%), and 50 ASA3 (43.4%). There was no significant difference in patient age nor in the ASA score for flap survival rates.

### Lesion Characteristics

One hundred and fourteen procedures were performed to reconstruct an oncological defect and one procedure was performed for reconstructive surgery in cleft palate sequel: 101/115 on squamous cell carcinoma (87.8%), 4/115 for basal cell carcinoma (3.5%), 6/115 (5.2%) primitive locally invasive bone tumors, one case of hemangio-pericythoma, oral floor adenocarcinoma, and neuroaesthesioblastoma, respectively.

Tumor was benign in 5/114 patients (0.5%), with local malignancy (N0 at the TNM classification) in 72/114 patients (63.1%) and with lymph node invasion (N+ at the TNM classification) in 37/114 patients (32.5%).

Nineteen procedures were performed on a surgical area that was irradiated preoperatively, and in addition to radiotherapy, two patients underwent chemotherapy, whereas one more patient was treated with neoadjuvant chemotherapy. There were 84/114 (73.7%) primary tumors and 30/114 (26.3%) recurrences/remnants.

### Reconstructive Details

The lesions involved the upper facial third in 4 patients (3.5%), the middle facial third in 24 patients (20.9%), and the lower third in 87 patients (75.6%) (Table 1).

**Table 1:** Flaps used for facial third reconstruction with the number of flaps re-explored and failed.

Flaps/facial thirds	RFF	FF	ALT	LD	DCIA	SF	GF	FC
Upper	1 (R1)	0	0	3 (R1)	0	0	0	0
Middle	6 (R1)	2	12	1	2 (R1,F1)	1	0	1
Lower	29 (R4,F2)	41 (R6,F7)	13	5	1	0	1	0

*RFF, radial free flap; FF, fibular flap; ALT, anterolateral tight flap; LD, latissimus dorsi flap; DCIA, deep circumflex iliac artery bone flap; SF, scapular flap; GF, gracilis flap; FC, medial femoral condile flap; R, revised; F, failed flap.*

#### Free Flaps

A double free flap was required in three wide defect reconstructions of the inferior facial third. Thus, we now present technical details and results over 118 free flaps. Among these series of flaps, we performed microvascular flap revision in 14/118 (11.9%). Our flaps failed in 10/118 (8.5%). We observed a significant association between flap revised and flap survived (*p* < 0.0001 at the Chi-square two-tailed test; OR = 0.0297; 95% CI 0.06274–0.1406). Flap microvascular re-exploration saved the flap in (7/14) 50%. Three flaps were lost without revision and two failed for arterial ischemia. One of them presented a delayed arterial ischemia at the seventh day post surgery. Among the five composite flaps with arterial failure, two fibular flaps (FFs) presented a partial sufferance of the skin. To elaborate, among our 10 failed flaps, we observed in 2 flaps (one with venous failure and one with an arterial failure) an initial partial loss of the cutaneous component, concomitant with surgical site infection (SSI).

The types of flaps selected were as follows: 43 (36.4%) fibular flaps (FFs) (6 were only bone flaps), 36 (30.5%) radial free flaps, 25 (21.1%) anterolateral tight flaps (ALT), 9 (7.6%) latissimus dorsi flaps, 2 (1.7%) DCIA, 1 (0.9%) scapular free flap, 1 (0.9%) femoral condyle free flap, and 1 (0.9%) gracilis free flap.

The type of flap chosen for each facial third defect is shown in Table 1. The most frequently used flap in the upper facial thirds is the latissimus dorsi myocutaneous flap, which is a large flap with a main pedicle, mostly used to cover cranioplasties. For the middle facial third, the ALT flap is more frequently used for its utility in skin coverage in the orbit and in the cheek. Its chimeric harvesting allows to fill the orbital and paranasal cavities. For the lower third, the fibular osteocutaneous flap is used most frequently, due to the necessity for mandible reconstruction. Most of our osteocutaneous FFs were harvested contralaterally first to have the flap pedicle oriented postero-caudally to reach more proximal recipient vessels in the neck, second to allow the skin coverage for the reconstruction of the oral mucosal defect, and finally, for the external skin defect coverage. On the contrary, when we had to reconstruct the condylar ramus as well, together with the mucosa, we used same-side harvesting.

#### Bony vs. Soft Tissue Flaps

We compared flap survival between bony flaps and soft tissue flap, and we found a significant difference between the two (*p* = 0.0067 *χ*^2^ two-tailed test; OR = 0.1413; 95% CI 0.02856–0.6991). We mostly used osteocutaneous flaps and only six purely bony flaps. We had a total of 47 bone flaps, and the break-up is as follows: 43 FFs, 2 DCIA, 1 scapular flap, and 1 femoral condyle free flap. Of our 10 failed flaps, 8 were bony flaps. In our case series, mandible reconstructions with fibular osteocutaneous free flaps were performed after a certain time point with in loco fibular shaping before cutting the vascular pedicle of the flap at the donor site (22/37), reducing the flap ischemia time (from a mean of 87–48 min); however, this did not significantly impact flap survival.

#### Previous Surgery

Flaps insetted for facial reconstruction on regions where surgery had already been performed in the past presented both a significantly higher revision rate and a lower survival rate (*p* = 0.0001; OR = 0.1005; 95% CI 0.02609–0.3870) and (*p* < 0.0001; OR = 106.7; 95% CI 25.79–441.1), respectively.

#### Previous Radiotherapy

We observed that radiotherapy before surgery did not influence our flap survival rate (*p-value ns*). However, among flaps needing revision, there was a higher prevalence of surgery on irradiated tissues (*p* < 0.0001; OR = 0.008398; 95% CI 0.001032–0.06837).

### Recipient Vessels and Grafts

#### Arteries

**Figure 3:**
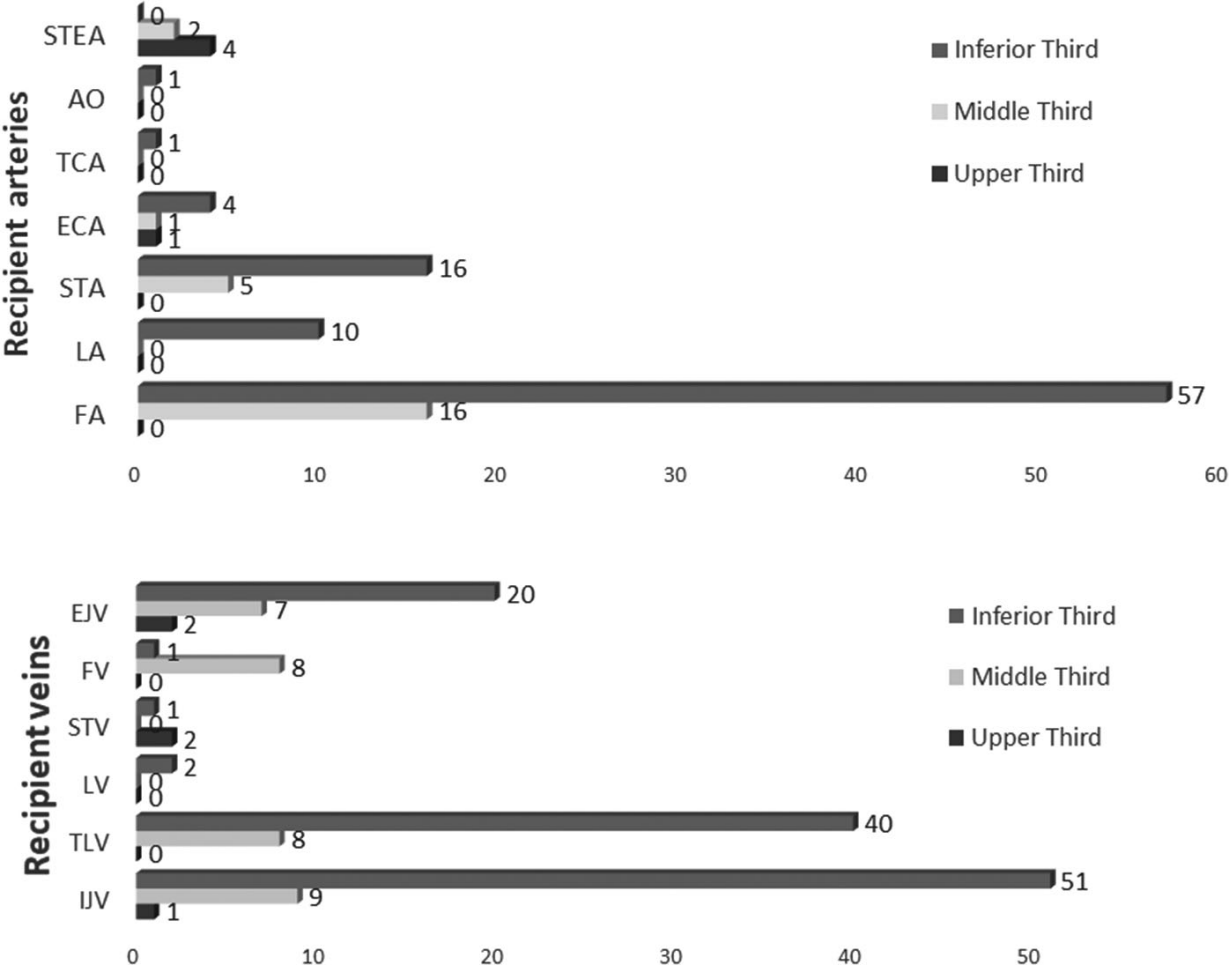
Recipient vessel choice for each facial third reconstruction. STEA, superficial temporal artery; AO, occipital artery; TCA, transverse cervical artery; ECA, external carotid artery; STA, superior thyroid artery; LA, lingual artery; FA, facial artery; EJV, external jugular vein; FV, facial vein; STV, superficial temporal vein; LV, lingual vein; TLV, thyro-linguo-facial trunk; IJV, internal jugular vein.

As regards the selection of the arterial supply, we privileged in the upper facial third the superficial temporal artery 4/5 (80%), for the middle third, the facial artery at its premandibular tract 16/24(66.7%), and for the lower facial third, again the facial artery, followed by the superior thyroid artery 57/89 (64%). In patients with a history of loco-regional radiotherapy (19/118 free flaps) before surgery, our recipient artery choice was conditioned, in that we performed anastomosis both as end-to-end and as end-to-side directly on the external carotid artery or contralaterally or on transverse cervical branches (TCA) from the succlavian artery system (Figure 3).

#### Veins

##### Double vs. Single Vein Drainage

For the venous anastomosis, we performed a double vein drainage for 47/118 (39.8%) flaps. There were 61/165 end-to-side anastomoses on the IJV. We observed that double vein flaps had no significant advantage either in terms of flap survival or in terms of flap revision (*p-value ns*).

##### Deep vs. Superficial Vein Drainage

We analyzed our flap series for deep (for double venous drainages at least one deep) 100/118 (84.7%) recipients or only for superficial venous 18/118 (15.3%) recipients. Also, this aspect apparently did not differ for the flap survival rate. However, there was a significant difference in the revision rate between flaps with only a superficial drainage and flaps with at least one deep venous drainage (*p* = 0.0246; OR = 3,846; 95% CI 1.115–13.27).

We had a deep venous recipient in 78.6% of single veins and in 93.8% of double veins with a significant difference (*p* = 0.0243; OR = 4.091; 95% CI 1.114–15.03).

The frequency of the specific vein recipient used is shown in Figure 3.

#### Vascular Grafts

We further investigated the possible implication of a graft interposition in the flap survival, and we observed that any graft for vascular anastomoses could be a sixfold risk for flap failure (*p* = 0.0023 OR = 6.714; 95% CI 1.721–26.19). In addition, we noted that grafts were associated with a more frequent re-exploration rate (*p* = 0.0037; OR = 5.25; 95% CI 1.1569–17.57). There was also a significant difference for arterial or venous pedicle grafts in relation to arterial or venous flap failure in particular (*p* < 0.0001; OR = 23.33; 95% CI 2.784–195.6) and (*p* = 0.0026; OR = 10.96; 95% CI 1.671–71.92), respectively.

An overview of the details of the above-mentioned features is illustrated for the failed flaps group in Table 2, and a general overview of the features associated with flap revision and flap survival is summarized in Table 3.

**Table 2:** Details of flap failure.

Facial thirds	Flap	Setting: in single or double flap recon.	Recipient artery	Recipient vein	Double vein	Deep vs. only superficial veins	Grafts	Vascular failure	Revised
Inferior	FF	Sg	FA	TLV	Sg	D	V	V	Y
Inferior	RFF	Sg	STA	IJV	Sg	D	No	V	Y
Inferior	FF	Sg	FA	TLV	Sg	D	V	A	Y
Inferior	FF	Sg	FA	EJV	Sg	S	A	V	N
Inferior	FF	Sg	FA	TLV	Sg	D	No	A	Y
Inferior	RFF	Sg	STA	TLV/EJV	Db	D	No	V	Y
Inferior	FF	Sg	FA	IJV	Sg	D	No	A	Y
Inferior	FF	Db	STA	IJV/IJV	Db	D	No	A	N
Inferior	FF	Db	FA	IJV	Sg	D	V	V	Y
Middle	DCIA	Sg	STA	FV	Sg	S	A	A	N

*FF, fibular flap; RFF, radial free flap; DCIA, deep circumflex iliac artery bone flap; Sg, single; Db, double; FA, facial artery; STA, superior thyroid artery; TLV, thyro-linguo-facial trunk; IJV, internal jugular vein; EJV, external jugular vein; D, deep; S, superficial; V, venous; A, arterial; Y, yes; N, no*.

**Table 3:** Summary of variables analyzed for flap revision and flap survival.

Flaps (total 118)	Not revised	Revised	(%), *p*-value * *χ*2 two-tailed test	Survived	Not survived	(%), *p*-value * *χ*2 two-tailed test
Total flaps	104	14	(11.9%)	108	10	(8.5%)
Revised						
No	–	–	–	101	3	*p* < 0.0001
Yes	–	–		7	7	
Previous surgery						
No	76	11	*p* = 0.0001	80	7	*p* < 0.0001
Yes	28	3		28	3	
Previous radiotherapy						
No	86	13	*p* < 0.0001	91	8	*p* = *ns*
Yes	18	1		17	2	
Flap components						
Soft tissue flap	64	7	*p* = *ns*	69	2	*p* = 0.0067
Bony flap	40	7		39	8	
Veins						
Double	43	4	*p* = *ns*	45	2	*p* = *ns*
Single	61	10		63	8	
Drainage						
Deep	90	9	*p* = 0.0246	92	8	*p* = *ns*
Superficial	13	5		16	2	
Grafts						
With any graft	13	6	*p* = 0.0037	14	5	*p* = 0.0023
Without grafts	91	8		94	5	
Excluding flaps with venous failure 5/118						
With graft for artery	3	2	*p* = 0.0121	3	2	*p* < 0.0001
With no graft for arteries	100	8		105	3	
Excluding flaps with arterial failure 5/118						
With graft for vein	13	3	*p* = *ns*	13	3	*p* = 0.0026
Without graft for veins	90	7		95	2	

## Discussion

The selection of arterial and venous recipients is the key in head and neck reconstruction (4–6).

**Figure 4:**
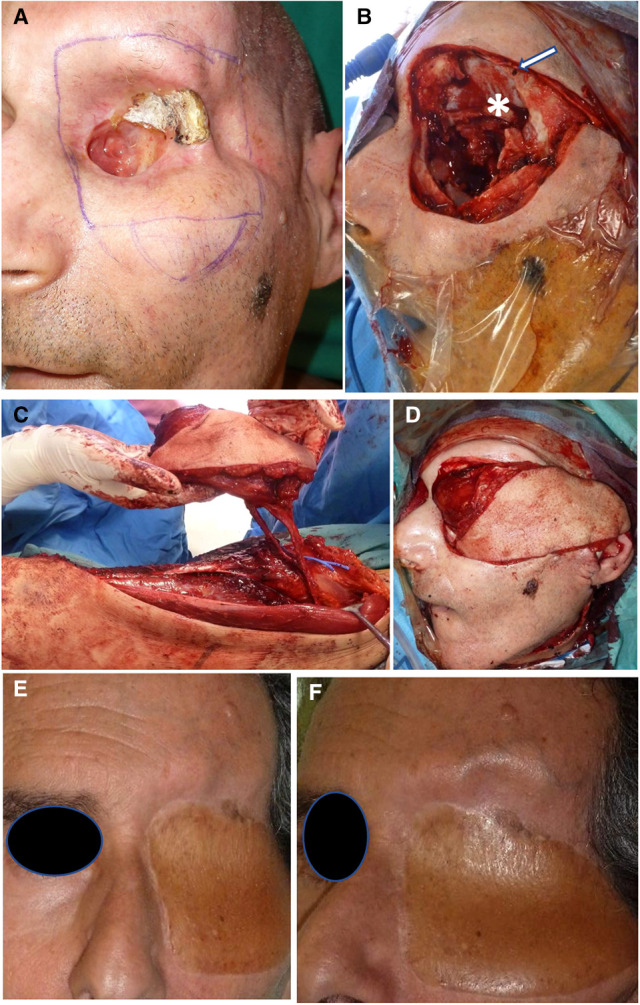
Orbital and skullbase resection and reconstruction. (**A**) Patient presenting with SCC remnant of orbital roof postradiation therapy and osteoradionecrosis. (**B**) Wide resection of the left orbit. Basofrontal dura is marked with (asterisk); dura suspension sutures (white arrow). (**C**) Chimeric ALT flap harvesting with vastus lateralis component. (**D**) Flap insetting and filling of the paranasal cavities with a vascularized muscle. (**E, F**) Frontal and side views of the final outcome at a 3-month follow-up.

Head and neck defects that require free flap reconstruction are related in most cases to squamo-cellullar tumor ablation. This accounts for several settings of surgery performed on tissues that had previous surgery or radiotherapy (7), see Figure 4.

Conventional flap monitoring (8) was useful to detect the need for revision surgery, especially for venous flap congestion. Arterial ischemia is sneakier and requires multimodal monitoring by trained personnel. Laser Doppler could improve preclinical detection of arterial insufficiency, saving time for the decision to go back to surgery for anastomosis re-exploration (9). Microvascular flap revision allowed us to save several cases of venous sufferance. Probably revision for arterial malfunctioning was performed too late to obtain flap salvage. According to the literature, the first 72 h are crucial for intensive flap monitoring, even though arterial complications can also occur after 1 week (8). The time lap from diagnosis of flap sufferance to surgical revision should be around 1 h to increase the likelihood of flap salvage (10). In the present series, bony flaps were more susceptible to failure than soft tissue flaps, as observed by other teams as well (11). Our ischemia time before and after the introduction of *in situ* modeling at our center did not influence flap survival, probably because both ischemia time ranges were within the average ischemia range required by other teams, ranging between 40 and 99 min (12, 13).

Our choice of recipient arteries depended on the type of flap pedicle length, facial third involved, previous surgery, or radiation therapy. We found that our preference was in agreement with that of other authors as regards the upper facial third: the superficial temporal artery (STEA) is the first choice followed by the distal facial artery (6). However, many studies analyze the middle and the inferior facial third reconstruction, together making a comparison more difficult (3, 6, 14); these authors use the superior thyroid artery as the first choice, followed by the facial artery. Instead, we used the facial artery as the first choice (Figure 2B), followed by the superior thyroid artery. Neck dissection often requires facial artery transection to remove the first cervical lymph node station, and using this artery avoids wastage of the facial artery already transected and maintains the superior thyroid artery as a future possibility or as a second choice if flow in the former (FA) is not satisfactory. Even in surgery where neck dissection is not required (basal cell carcioma recurrence), the recipient vessels are harvested from the premandibular tract of the facial vessels (15).

Radiotherapy or previous surgery did influence our vessel choice: arteries can be atrophic, the arterial wall can be fragile and rigid, the vessel lumen of the carotid branches present stenoses and insufficient flow, and their dissection within scar tissue can be more troublesome and dangerous. Thus, we followed the ECA branches from distal to proximal evaluating intraoperatively to perform anastomosis either on an emergent branch from the ECA or an end-to-side anastomosis on the ECA (Figure 2C). In some cases, an end-to-end anastomosis with an atrophic ECA is a possibility. Going contralaterally or down to the TCA increases the likelihood of needing a graft. However, some authors select the same side TCA, avoiding a graft by choosing a flap provided with a long pedicle (16).

Some authors from their experience found that presurgical radiation therapy could impact flap survival (17). We avoided this inconvenience, preferring either main and long pedicle flaps or a perforator flap with an eccentric skin paddle or FFs with a more distal (caudal) bony component to gain pedicle length. However, presurgical radiation affected our flap revision rate. Previous surgery can be considered as a risk factor in our series for a greater likelihood for re-exploration and for flap survival.

The controversy surrounding the advantage of the second vein and the skepticism of plastic surgeons with a background in limb reconstruction can be explained as follows: the outflow velocity of the same flap is lower when implanted on the limb than on the head and neck region (18). This explains the preference for a single vein in limb reconstruction to avoid too low outflow velocity and following an increased risk of thrombosis. In flaps draining into the central venous system, the flow reduction provided by the second vein does not reach the threshold for thrombosis because of a higher negative pressure driving forces (19). These observations, as discussed in a meta-analysis (20), account for different microvascular dynamics between limb and head and neck.

A recent meta-analysis (20, 21) concludes that a double vein anastomosis is likely to increase the flap survival rate. Our study does not confirm the advantage over flap survival or flap re-exploration rate provided by the second vein.

We searched for an explanation for this different result from our study. We observed that the flap revision rate was lower if there was at least one deep venous recipient vessel.

**Figure 5:**
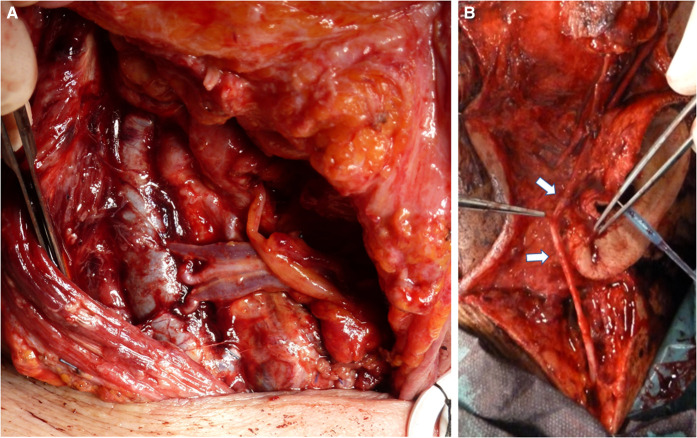
Venous recipient vessel. (**A**) End-to-side double vein anastomosis between the internal jugular vein and the comitant veins of the gracilis flap pedicle. (**B**) Anastomosis with graft (white arrow) to the retromandibular tract of the external jugular vein freed from the parotid.

This was also found in one of the two meta-analyses, in which we observed that anastomosis in the IJV system is safer than the superficial recipient veins (21), but this is not univocal (22). In our practice, we aim at having at least one deep venous recipient where possible. Deep veins as recipients have the advantage of a better flow, as well as a lower risk of direct compression. We noted that most of the deep vein anastomoses had been performed as end-to-side on the IJV in order to avoid vessel kinking by the possibility of choosing the best pedicle position along the cranio-caudal IJV axis for anastomosis (Figure 5A). In the group of single vein anastomoses, especially for the reconstruction of the upper and middle face compartment, the distance to reach the deep venous system could be a limiting factor.

In general, graft interposition to reach recipient vessels can be associated with an increased risk of flap loss (5%–35% vs. 1.1%–7% without grafts, as revised in 23). This view is not shared by all (7).

For patients with a history of radiotherapy or previous surgery, we considered the issue of the vascular pedicle’s length for selecting a safe flap to possibly avoid grafts, if the same reconstructive quality can be maintained. Grafts for arterial pedicle elongation were used in these patients mainly for the inferior third or for the middle third reconstruction when there were no alternatives.

Intraoperatively, when proper potential flow efficiency was doubtful, we changed our strategy and opted for a graft interposition to reach a safer vessel (Figure 5B). Our grafts are mostly used to elongate venous pedicles for the second vein drainage.

## Conclusion

During neck lymph node dissection, the facial artery has to be carefully prepared as a first choice recipient, and in order to save the superior thyroid artery, as a second option, and/or for further microsurgical procedures. At least one of the venous anastomoses should target the internal jugular venous system. Our thinking on the venous outflow was favorable for double veins even if a graft was required for the second vein. However, the analysis of our series discourages the use of grafts. Therefore, flap and recipient vessel choice should avoid graft interposition. Flap harvesting should preserve the maximum pedicle length. For upper and middle facial third reconstruction, a graft could be unavoidable, considering esthetic and functional requirements in a complex anatomical region.

Flap monitoring to detect arterial insufficiency should be meticulous and prolonged over 1 week.

## Data Availability

The raw data supporting the conclusions of this article will be made available by the author PI upon motivated request.
